# Successful captive breeding of vultures due to the double clutching method

**DOI:** 10.3897/BDJ.12.e126082

**Published:** 2024-08-16

**Authors:** Rusko Petrov, Andreana Dicheva

**Affiliations:** 1 Green Balkans - Stara Zagora NGO, Stara Zagora, Bulgaria Green Balkans - Stara Zagora NGO Stara Zagora Bulgaria; 2 Trakia University, Stara Zagora, Bulgaria Trakia University Stara Zagora Bulgaria

**Keywords:** Cinereous Vulture, *Aegypiusmonachus*, Griffon Vulture, *Gypsfulvus*, captive breeding, raptor conservation

## Abstract

After a massive decline, the Griffon Vulture (*Gypsfulvus*) population in Bulgaria has now stabilised as a result of the hard work of conservation programmes, although it is still listed as an Endangered species (EN) in the country's Red Data Book. Due to a series of unfortunate events, another species - the Cinereous Vulture (*Aegypiusmonachus*) became extinct in Bulgaria and recovered only recently due to systematic re-introduction efforts. Along with the poor living conditions, a predisposing factor for the decreasing population of the vultures is the fact they hatch only a single egg; two may be laid on exceptions. The survival rate of the young is from medium to low. In that order, a method of double clutching has been applied in the hope of preserving and retrieving both of the vultures. This article aims to introduce the double clutching method in detail, as well as to show the results of its implementation into the breeding programme of the Cinereous Vulture and the Griffon Vulture at the Wildlife Rehabilitation and Breeding Centre of Green Balkans – Stara Zagora NGO. This research studies the time period 2019-2023. The purpose of this study is to compare the results of natural copulation and hatching and those of the double clutching method. The data taken are used for studies for both the Cinereous and the Griffon Vulture’s reproduction in correlation with their feeding programme and living conditions. The results of the study will be summarised into the following text for the purposes of shedding light on the positives of the double clutching method.

## Introduction

### Status

The Griffon Vulture (*Gypsfulvus*) has been declared extinct, but in the 70s of the 20^th^ century, a small colony has naturally returned as nesting in the Eastern Rhodope Mountain (Michev et al. 1980). Due to decades of conservation activities, this last remaining colony, located at the region of Studen kladenets, has been significantly boosted and the species has been re-introduced also into several other territories around the country - Sinite Kamani Nature Park, Kotel Mountain, Vrachanski Balkan Nature Park and Kresna gorge in Pirin Mountain, amounting to around 100 pairs in 2016 (Stoynov et al. 2018). The Griffon Vulture is currently listed as an Endangered species in the Red Data Book of the Republic of Bulgaria (Yankov et al. 2015).

Only a decade of poor conditions were needed for the Cinereous Vulture (*Aegypiusmonachus*) to be classified as an extinct species (EX) in the Red Data Book of the Republic of Bulgaria (Marin et al. 2015) . Once widespread (late 19^th^ and early 20^th^ century), the Cinereous Vulture was lastly observed in 1993, in Eastern Rhodope Mountain (Marin et al. 1993), when an actively breeding pair was found, but just for a single season. Until recently, the only remaining colony of this species on the Balkans was located in the Greek part of the Eastern Rhodope Mountain, at the National Park of “Dadia”. From 2021, the Cinereous Vulture has been established as a breeder once again in Bulgaria - in Stara Planina mountain, thanks to systematic releases of birds since 2018 (Ivanov et al. 2024). The species is protected by Annexes II and III of the Bulgarian Biodiversity Act (2002) and is categorised by the IUCN as Near Threatened (NT) (BirdLife International 2021).

### Threats

A series of events, primarily anthropogenic, brought the species to a critical point, which, in its turn, concentrated the attention and efforts of a number of Bulgarian NGOs and institutions on the vulture conservation issue. With the pastoral transhumance being progressively abandoned, the presence of ungulate herds in the highland areas - key source of food for the vultures (Olea and Mateo-Tomás 2009), became scarce and has gradually been demolished. The unreasonable belief that vultures are birds of prey, once widespread amongst those unaware of the biology of the scavengers, especially in the rural areas, forced farmers and hunters in vulture regions to intensively hunt on vultures in order to protect their farm animals from attacks. A significant historic event, coinciding not only with the decrease of the number of vultures, but also of other species of birds such as the Common buzzard, Golden eagle, Goshawk etc., took place during World War II. Back then, the well-known insecticide - Dichlorodiphenyltrichloroethane (DDT) was introduced and widely used (Mirmigkou and de Boer 2015), causing ecological disaster for vultures and other species in many countries. All these and some other threats with lesser or greater impact, have initiated the implementation of a series of activities, aiming to monitor, conserve, re-introduce or boost the population of the vulture species in Bulgaria (Nikolova 2010).

### Double clutching

Courtship in the Griffon Vultures pair usually started in late November with nest building and first copulations. The breeding season of the Cinereous Vultures usually started in November with nest building and allopreening. First copulations are observed in February, but they can occur even in January as the birds become more experienced. In the wild, Griffon and Cinereous Vulture pairs normally produce only one egg per year (Gómez-López et al. 2023). In case of accidental loss of the egg early in the season, the birds can naturally double-clutch and produce a replacement egg (Martinez et al. 1998). The second egg can be laid only if mating starts again and the breeding season has not yet finished. Following this natural behaviour, captive-bred pairs of various species are often given the chance to achieve double success in reproduction - by removing the laid egg and incubating it artificially as the pair lays and incubates the second one. A number of factors impact the process: quality and quantity of the food, preventing unnecessary stressors and proper housing conditions (Newton 1979).

This study describes the process of inducing double clutching in order to maximise the breeding output of Griffon and Cinereous Vultures breeding in captivity in Bulgaria. The research was conducted in the Wildlife Rehabilitation and Breeding Centre (WRBC), part of Green Balkans - Stara Zagora NGO, in Bulgaria and, potentially, the methods and outcomes could benefit other similar facilities and breeding programmes for vultures.

## Material and methods

Two captive-bred pairs - one of Griffon and one of Cinereous Vultures, have been included in the breeding programme in the WRBC intended to increase the number of the local population by producing parent-reared chicks which subsequently are released into the wild. Some of the birds in the pairs were replaced over the years; however, all were disabled and incapable of surviving in the wild.

The male Griffon Vulture B58 was caught in the wild in Bulgaria exhausted and in a generally bad condition. At the end of 2006, it was admitted into the breeding programme of the WRBC. Its first partner was the vulture Leshka. In 2014, the pair had their first chick. This was followed by two unsuccessful breeding seasons after which the female laid unfertilised eggs. The couple were separated in 2016. B58 was moved to the visitors’ part of the Centre. The female Griffon Vulture K8X arrived in June 2014 from Spain where it was captured from the wild. In Bulgaria, it was released from the aviary in Kotel. In December 2014, it was found shot in Balabankuru, Turkey and was treated in the Faculty of Veterinary Medicine of Istanbul University. It was operated on to remove the bullet and, as a result, a joint in her left wing was blocked. In December 2015, K8X was admitted in the WRBC to become part of the breeding programme with the male KOJ; however, the male showed aggression and the pair were separated. A new pair was formed - B58 and K8X and their first successful season was in 2019.

The first Cinereous Vulture in the breeding programme of the WRBC was admitted in 2011. It was the female with ring number J2028 which arrived from Spain after being rescued from the wild. It was hatched in 2009. In 2014, a male bird arrived from France with ring number A19, hatched in the wild. The pair were separated at the end of 2018 because they were unsuccessful as they never copulated or had fertile eggs. The female was given a chance with another partner in 2019. The male was replaced with a bird from Ouwehands Dierenpark Zoo in the Netherlands - ring number CS, hatched in 2012. All their eggs were either infertile or died during incubation due to excessive weight loss. In 2023, the female was changed with another disabled bird - ring number K2. K2 was captured in 2017 in Spain and had partial amputation of one wing.

### Interior of the cages

**Figure 1.**
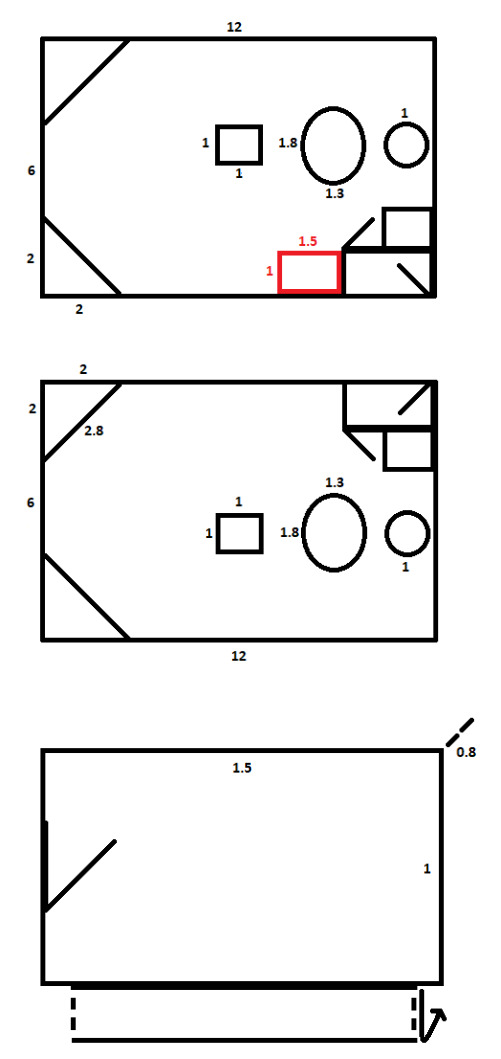
Measurements of the cages for Griffon and Cinereous Vultures in the WRBC.

The cages of the vultures have been constructed suitable for the purposes of the captive breeding: 12 metres long, 6 metres wide and 4 metres high (Fig. 1). They provided the birds enough space to easily perform short flights and jumps within the cage. The door is on the opposite side of the nesting platforms, preventing the vultures from stress when keepers enter the cage for cleaning, changing water or reconstructions. The cage has two water ponds: one larger oval-shaped (1.8/1.3m) and a smaller spherical one (1/1m) with a tap water system, so the birds have access to fresh water for drinking and bathing. There is a feeding platform on the ground beside the door (1/1m) and also a feeding table installed in the middle of the cage. The two corner nesting platforms (2/2/2.8m) are at 2.7 metres height from the ground, with four stairs for climbing. Two of the walls and the roof are with mesh giving the birds sight to the surroundings. The double-door protection is a crucial safety precaution to prevent the birds from escaping the cage when opening the inner door. Food is provided through a small door in the corridor to keep entrance of the breeders in the cage to a minimum - once a week for cleaning of the cage and water ponds and twice a year for medical examination of the birds. Thorough cleaning and disinfection of the facilities is made twice a year during the regular health examination of the pairs when the birds are taken out to avoid stress. The following was the protocol described in a publication by Huyghe et al. (2023).

### Menu

Food was provided on a daily basis in rations of 400 grams per bird with one fasting day weekly (Table 1). The menu included lamb/goat meat twice a week, rabbit twice a week, horse/beef twice a week. During the breeding season, rats and rabbits are given twice a week and, once a week, lamb/goat and horse/beef. In cold weather or at the beginning of the breeding season, the amount of food can be doubled to avoid conflicts amongst the birds. The fasting day can also be skipped if aggression is observed.

### Video surveillance

Two surveillance cameras were installed in each cage – one focused on the nesting platform and one with a general view of the cage. Standard fixed security IP cameras (Dahua) were mounted with a resolution of 2-8 megapixels that recorded any movement 24/7 to standard NVR devices. The video records were kept for 30-45 days depending on the season. The cameras had live view, playback and video and photo download options.

### Behaviour and breeding methods

The Griffon Vulture nest was built by the keepers at the WRBC, but the birds were given the chance to enhance it by using the nest material regularly provided in the cage. When the egg has had at least ten days of natural incubation, it was removed from the nest, candled and artificially incubated until the end of the incubation period. The incubator used was Masalles Falcon C30-S. The second egg was left to be naturally incubated as the parents had experience. The first egg was kept in the incubator for 57 days and, if it did not hatch, it was opened and checked. If hatched, the chick was given to the parents after at least 7 days of hand rearing and then the second egg was taken to the incubator - at temperature of 36.6°C and humidity of 40%.

The weight loss of the egg was strictly being monitored. The same procedure followed with the second egg and, if it hatched, it was not adopted by the pair, being unnatural for the Griffon Vultures to raise two chicks in one clutch. In such cases, an adaptation box had to be constructed and placed within the main cage within visual contact of the parents. The box was 1.5/1/0.8 m with a small feeding opening on the wall connected with the corridor and a larger door on the wide side of the cage for inserting the chick. Again after 7 days of hand rеaring, the second chick was introduced to the parents and the first chick was taken from the nest and placed in the adaptation box. At this age, the first chick was able to thermoregulate and feed independently. When it changed its plumage, it was transported to an adaptation aviary. The same procedure followed with the second chick.

For the Cinereous Vultures, concerning their history of breaking eggs, when laid it was immediately taken to the incubator. The second egg was also artificially incubated. The pair was given a dummy egg in order to stimulate them to incubate and prepare for adopting a chick. Both eggs were incubated at 37°C and starting humidity of 45%. To prevent weight loss, humidity was slowly increased until the excessive evaporation regulated.

## Results

The data for the first pair are summarised in Table 2,and for the second - in Table 3.

**Figure 2.**
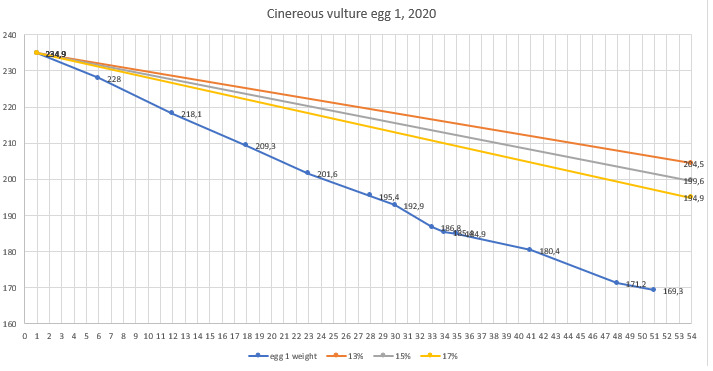
Season 2020 - egg 1 weight loss.

**Figure 3.**
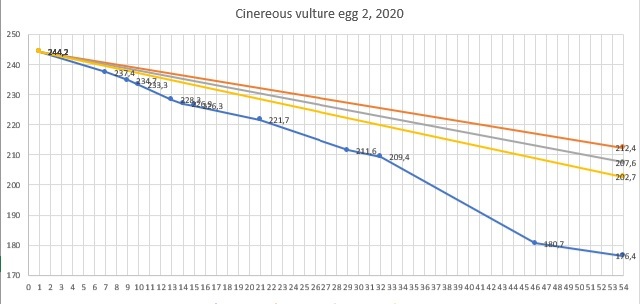
Season 2020 - egg 2 weight loss.

**Figure 4.**
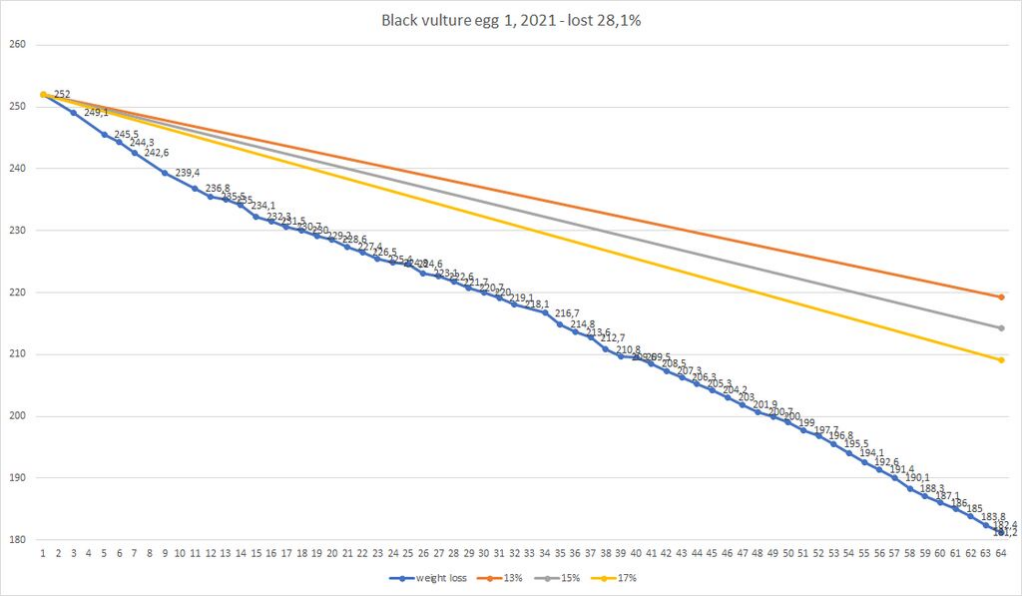
Season 2021 - egg 1 weight loss.

**Figure 5.**
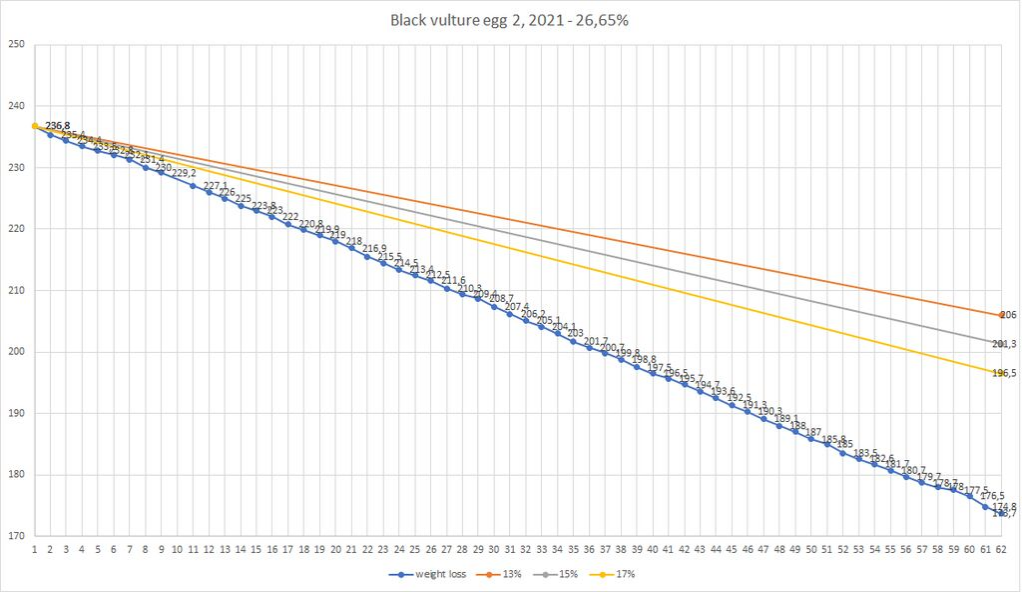
Season 2021 - egg 2 weight loss.

**Figure 6.**
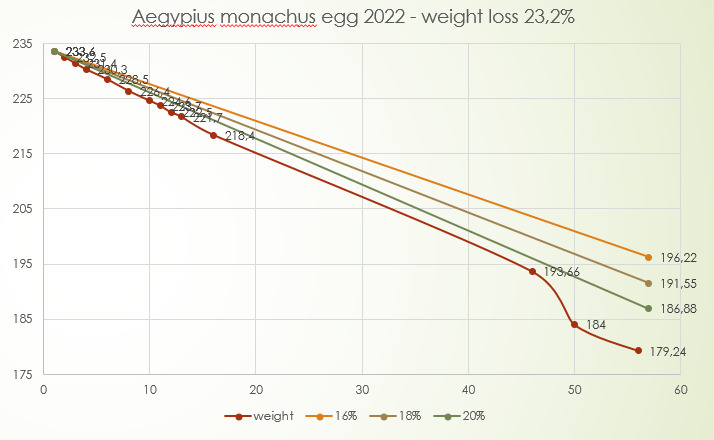
Season 2021 - egg 1 weight loss.

**Figure 7.**
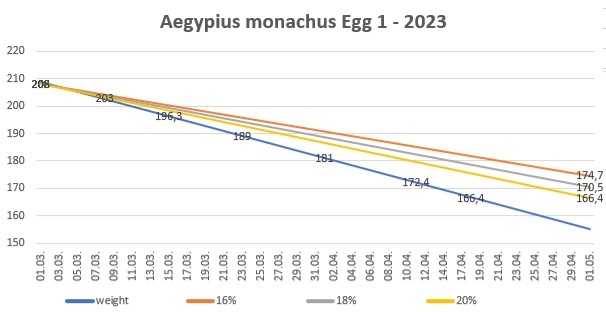
Season 2023 - egg 1 weight loss.

**Figure 8.**
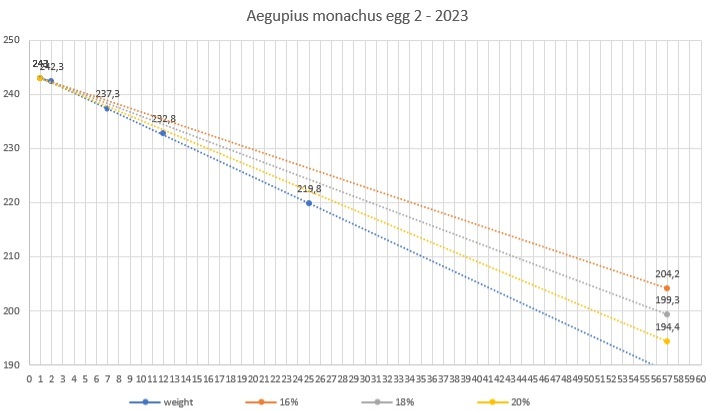
Season 2023 - egg 2 weight loss.

The first Cinereous Vulture pair had their first egg laid on 28/3/2017, but it was infertile as they never had experience or copulations. The female was also not experienced in incubating and she broke it on the next day. Next season, on 9/4/2018 she laid an egg, which again was infertile and she broke it after one day. The pair was separated at the end of 2018 and the female was given a chance with another partner. The second pair started copulating and their first egg was hatched on 31/03/2019. On the next day - 01/04/2019, it was taken to the incubator and artificially incubated for 58 days. It was opened on 29/05/2019 and was found infertile. In season 2020, they started copulating on 19/03/2020. The first egg was laid on 25/03/2020 and immediately taken to the incubator. On 30/03/2020, they started copulating again. Second egg was laid on 15/04/2020 and taken to the incubator on 16/04/2020. The first egg was opened on 22/05/2020 and the second egg was opened on 10/06/2020. Both eggs were found infertile. From 2020, the weight loss of the eggs was monitored (Figs 2, 3, 4, 5, 6, 7, 8).

The first copulations for 2021 occurred on 29/01/2021 and the first egg was laid on 31/03/2021. On 01/04/2021, it was taken to the incubator. After three days - on 04/04/2021, the pair started copulating again and the second egg was laid on 24/04/2021. The first egg was opened on 02/06/2021 and the second - on 24/06/2021.

In season 2022, the first copulations occurred on 21/01/2022. The first egg was laid on 04/04/2022 and taken to the incubator on the same day. It was artificially incubated until 31/05/2022. It was infertile, but the embryo had died during incubation after weight loss of 23.2%.

In season 2023, they double-clutched again. The first copulation was spotted on 17/01/2023 and the female laid on 03/01/2023. Two days later - on 03/03/2023, the egg was moved to the incubator and it was opened on 04/05/2023. The second egg was laid on 06/04/2023 and taken to the incubator on the same day. It was opened on 01/06/2023. Both eggs were fertile, but the embryos had died during incubation. All data were systematically entered into a digital database (Table 4).

The days between each step of the process of breeding varied from one year to another. Differences were also observed between the clutching periods of the Griffon and the Cinereous Vulture pairs (Tables 5, 6). For the Griffon Vultures, it took 14 days from the day of copulation to the day of laying the egg of the first clutch in 2020. Meanwhile for the same year, in the Cinereous Vultures’ nest, this period was only 6 days. One year later, that same period took much longer for both of the pairs. The Griffon Vultures laid their egg 65 days after their first copulation was detected and, similarly, 61 days were needed for the Cinereous Vultures. In 2022, this period was shorter for the Griffon Vultures - 38 days, while it took 73 days for the Cinereous Vulture from the first copulation to the laying of the egg. The day gap for the period counted for the second clutch is less variable. In 2020 for the Griffon Vultures pair, it took 25 days and for 2021 - 24 days, likewise for the Cinereous Vultures’ couple in 2020, this period was 16 days and, for 2021, 20 days. For the Griffon Vulture, the first laid egg in 2020 was taken from the pair on the 17^th^ day after the laying and, in 2021, it was taken on the 14^th^ day and a second clutch was successful for both in this year. What is interesting is that, in 2022, the egg from the first clutch was taken from the parents on the 13^th^ day, but the attempt for the second clutch was not successful. The days that were needed for the egg of the Griffon Vulture to stay in the incubator were 42 for 2020 and 40 for 2021. For the second clutch in 2021, the egg stayed in the incubator for 34 days. Meanwhile for the first clutched egg of the Cinereous Vultures, it took much longer- 58 days for 2020 and 63 days for 2021. The second one stayed in the incubator for 55 days for 2020 and 61 days for 2021. The year of 2021 is indicative for the development of the two Griffon Vulture chicks. The first one was raised in the incubator for 6 days and then was given to the parents who took care of it for 68 days. The second chick was raised by the parents for 109 days. Looking back in 2020, this period was 125 days for the chick from the second clutch. The first chick was separated from the parents at the age of 74 days and the second one at the age of 122 days.

## Discussion

Standard protocols regarding breeding the vulture species in captivity were followed (Prakash 2012, Wolter et al. 2015). Our study involved one pair of Griffon Vultures and one pair of Cinereous Vultures - with some changes in the partners through the years, breeding in captivity in the WRBC in Bulgaria. A second clutch was induced on six occasions for the Griffon Vulture pair and on three for the Cinereous Vulture pair. The time between the taking of the first egg and the laying of the second one was approximately 27.5 days for the Griffon and 26.3 days for the Cinereous Vultures in our study. This corresponds to the interval of 20-28 days and results from other studies both of wild and captive vultures (Mendelssohn and Leshem 1983, Martinez et al. 1998). Renesting is often associated with increased breeding success in various bird species (Morrison 1998, Monroe et al. 2008 , Jackson and Cresswell 2017, De la Cruz et al. 2022) at the same time; for Griffon Vultures and others - a lower fledging success has been estimated for hatchlings from second clutches compared to those from the first, due to late fledging dates (Linden and Møller 1989, Brouwer et al. 1995, Martinez et al. 1998, Casas et al. 2009). On the other hand, in facilities where breeding and release happen in a controlled environment, this downside is avoided and, in turn, the second offspring of raptors is positively related to increased re-introduction and conservation success (Wood and Collopy 1993, Khan and Murn 2011, Bowden et al. 2012).

In the WRBC, the overall breeding success for the Griffon Vultures was aided by the renesting - from all the first clutches between 2013 - 2022, there would have been only one reared chick, compared to four reared in total from both clutches for the same period. This showed the positive effect of inducing renesting - even a few individuals can be of great importance to conservation programmes, which justified the efforts. For the Cinereous Vultures, double clutching made no difference to the breeding success due to the lack of successful hatchings from any of the clutches to date - this poses a need for further investigation in order to determine the cause of the problem.

## Figures and Tables

**Table 1.** The menu for Griffon and Cinereous Vultures in the WRBC.

**Menu during the breeding season**	
Monday	rat
Tuesday	rabbit
Wednesday	horse/beef
Thursday	rat
Friday	lamb/goat
Saturday	rabbit
Sunday	fasting day
**Menu outside the breeding season**	
Monday	lamb/goat
Tuesday	rabbit
Wednesday	horse/beef
Thursday	rabbit
Friday	lamb/goat
Saturday	horse/beef
Sunday	fasting day

**Table 2.** Data on pair B58 / W0104 at the WRBC - first and second clutches.

B58 / W0104	First egg	Hatched	Second egg	Hatched
2013	18/1/2013	-	21/3/2013	-
2014	27/1/2014	-	3/3/2014	29/4/2014
2015	19/1/2015	-	22/2/2015	-
2016	9/1/2016	-	8/4/2016	-

**Table 3.** Results from the Griffon Vultures B58 / К8X’s first and second clutches.

**First clutch**	First copulation	Egg laid	Taken to incubator	Hatched	Hatched in incubator	Chick introduced to the parents	Chick separated from the parents
2019	6/1/2019	18/1/2019	-	19/3/2019	-	-	-
2020	16/12/2019	30/12/2019	16/1/2020	27/2/2020	-	-	-
2021	20/10/2020	24/12/2020	7/1/2021	-	16/2/2021	22/2/2021	1/5/2021
2022	20/11/2021	28/12/2021	10/1/2022	-	19/2/2022	2/3/2022	6/6/2022
**Second clutch**	First copulation	Egg laid	Taken to incubator	Hatched in incubator	Hatched in the nest	Chick introduced to the parents	Chick separated from the parents
2020	20/1/2020	14/2/2020	-	-	7/4/2020		10/8/2020
2021	9/1/2021	2/2/2021	22/2/2021	28/3/2021	-	10/4/2021	28/7/2021

**Table 4.** Results from the Cinereous Vultures’ first and second clutches.

**First clutch**	First copulation	Egg laid	Taken to incubator	Egg opened
2019	-	31/3/2019	1/4/2019	29/5/2019
2020	19/3/2020	25/3/2020	25/3/2020	22/5/2020
2021	29/1/2021	31/3/2021	31/3/2021	2/6/2021
2022	21/1/2022	4/4/2022	4/4/2022	31/5/2022
2023	17/1/2023	1/3/2023	3/3/2023	4/5/2023
**Second clutch**	First copulation	Egg laid	Taken to incubator	Egg opened
2020	30/3/2020	15/4/2020	16/4/2020	10/6/2020
2021	4/4/2021	24/4/2021	24/4/2021	24/6/2021
2023	4/3/2023	6/4/2023	6/4/2023	1/6/2023

**Table 5.** Data for the Griffon Vultures in days.

**First clutch**	Copulation to hatching	Incubating by parents	Incubating in incubator	Incubation period	Raising in the incubator	Raising by the parents	Separating from the parents
2019	12	60	-	-	-	-	-
2020	14	17	42	-	-	-	-
2021	65	14	40	54	6	68	74
2022	38	13	11	53	11	96	107
**Second clutch**	Copulation to hatching	Incubating by parents	Incubating in incubator	Incubation period	Raising in the incubator	Raising by the parents	Separating from the parents
2020	25	53	-	53	-	125	125
2021	24	20	34	54	13	109	122

**Table 6.** Data for the Cinereous Vultures in days.

**First clutch**	Copulation to hatching	Incubating by parents	Incubating in incubator
2020	6	-	58
2021	61	-	63
2022	73		57
**Second clutch**	Copulation to hatching	Incubating by parents	Incubating in incubator
2020	16	1	55
2021	20	-	61
2023	33	-	56
